# Dental Impaction in the Cecum: Case Report and Review of Gastrointestinal Foreign Body Impactions

**DOI:** 10.1155/2018/2154879

**Published:** 2018-06-10

**Authors:** Mouhanna Abu Ghanimeh, Omar Abughanimeh, Sakher Albadarin, Osama Kaddourah, John H. Helzberg

**Affiliations:** ^1^Henry Ford Health System, 2799 W Grand Blvd, Gastroenterology K-7 Room E-744, Detroit, MI 48202, USA; ^2^University of Missouri-Kansas City, School of Medicine, Internal Medicine-Graduate Medical Education, 2411 Holmes Street, M2-302, Kansas City, MO 64108, USA; ^3^Saint Luke's Hospital of Kansas City, Division of Gastroenterology, Mid-America Gastro-Intestinal Consultants, 4401 Wornall Rd, Kansas City, MO 64111, USA; ^4^University of Missouri of Kansas City/School of Medicine, 2411 Holmes Street, Kansas City, MO 64108, USA; ^5^Saint Luke's Hospital of Kansas City, 4401 Wornall Rd, Kansas City, MO 64111, USA

## Abstract

Approximately 20% of the adult population in the United States wears dentures. Foreign body ingestions, including dentures, are not uncommon. Although the majority of all ingested foreign bodies pass spontaneously through the gastrointestinal tract, impaction may occur, especially with physiologic constrictions, angulations, or stenosis. The esophagus is the most common site of impaction, whereas colonic impaction is extremely uncommon. We present a case of an 84-year-old male who was referred to the gastroenterology clinic for denture impaction, which lasted for two weeks. The patient had already failed to pass the denture following conservative treatment with laxatives, and repeated abdominal imaging showed the dental plate in the cecum. Colonoscopy was performed three weeks after the ingestion of his dentures, and tripod forceps were used to dislodge the end of the dental plate and ultimately remove it. The patient was asymptomatic for the entire period.

## 1. Introduction

Foreign body ingestion is not uncommon. It is more common in children and males [1–7]. In addition, certain factors increase the risk of foreign body ingestion, including extreme age, edentulism, maxillofacial trauma, psychoneurological deficit, and impaired sensorium [1, 8, 9]. Approximately 80% of all ingested foreign bodies, including dentures, pass spontaneously through the gastrointestinal tract [3, 4]. However, impaction with physiologic constrictions, angulations. or stenosis is possible [10–12]. The esophagus is the most common site of impaction. In contrast, impaction in the small and large bowel is far less common [5, 9, 10]. The clinical presentation of foreign body impaction depends on the site of impaction and the presence of complications. Three management modalities have been described for foreign body ingestion and impaction: observation (wait and watch), endoscopy. and surgery [1]. Perforation, penetration of adjacent organs, bleeding, and obstruction are reported complications that warrant urgent surgical intervention [13–16]. Fortunately, less than 1% of all cases require surgery [17].

## 2. Case Summary

An 84-year-old male was referred to the gastroenterology clinic due to colonic foreign body impaction. The patient reported that while eating a nectarine two weeks prior to his clinic visit, he believed that he had inadvertently swallowed his partial denture. The patient was asymptomatic.

**Figure 1:**
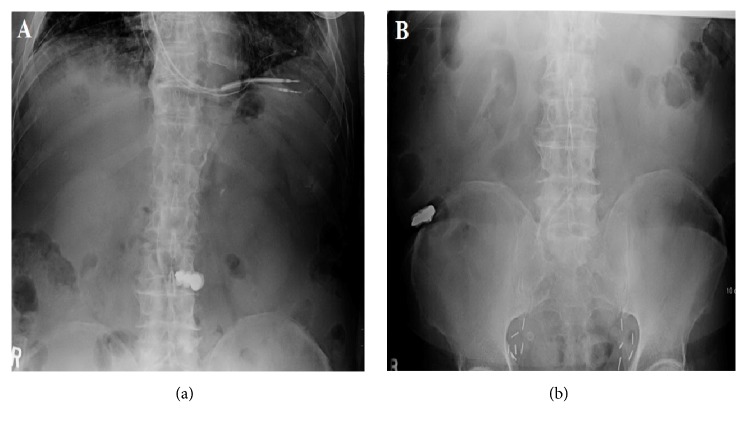
(a) The first abdominal radiograph demonstrating the dental plate in the mid abdomen. (b) The second abdominal radiography demonstrating the dental plate in the right lower quadrant and likely in the cecum.

Prior to his referral to the gastroenterology clinic, the patient's primary care physician had managed him conservatively with laxatives and obtained two abdominal radiographs to document the possible passage of the denture. The first radiograph showed the dental plate in the mid abdomen [Figure 1(a)]. The second radiograph revealed that the dental plate was in the right lower quadrant and likely in the cecum [Figure 1(b)].

**Figure 2:**
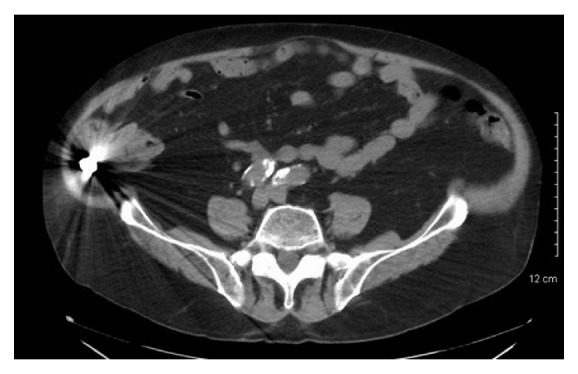
A CT abdomen and pelvis without contrast demonstrating the dental bridge persistently in the base of the cecum.

A CT scan of the abdomen and pelvis was obtained to determine the exact location of the dental plate (cecum or terminal ileum) and showed it at the base of the cecum [Figure 2].

**Figure 3:**
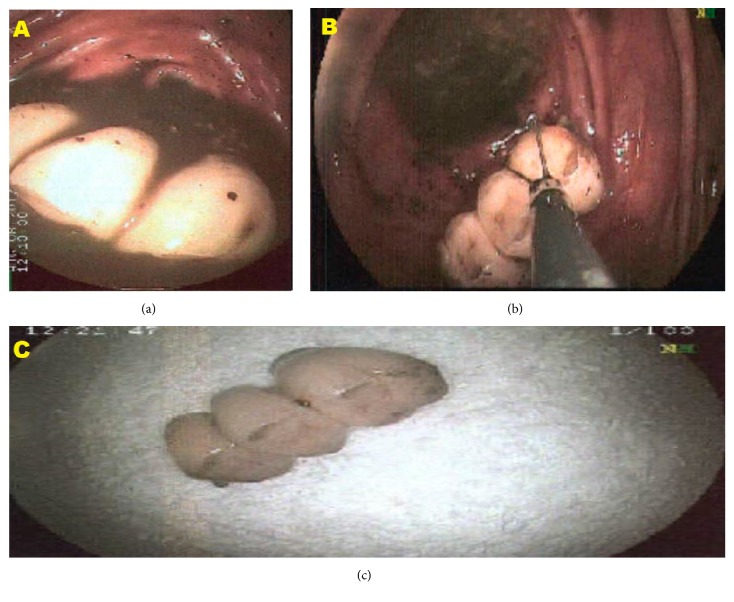
(a) Dental plate impacted in the cecum. (b) A colonoscopy forceps was used to grasp the end of the dental plate and to extract it. (c) The dental plate after extraction.

As the patient failed to pass the denture following conservative management with laxatives, the decision was made to attempt endoscopic removal. Colonoscopy was performed three weeks after the ingestion and showed that the dental plate had embedded in the cecal wall by a wire. The appearance was that of a face with smiling teeth. Tripod forceps were used to dislodge the end of the dental plate. It was then removed without difficulty or complication (Figures 3(a)–3(c)).

## 3. Discussion

Dentures are medical prostheses that are used to improve the mastication, articulation, and even self-esteem of people with poor dental or oral conditions. [1] Approximately 20% of the adult population in the United States wears dentures [1]. Denture ingestion is considered to be a multidisciplinary problem [1]. Gastroenterologists have an important role in its diagnosis and management; however, dentists, surgeons, and otolaryngologists play a crucial role in some cases.

Foreign body ingestion, including dentures, is not uncommon. The type of foreign body as well as the clinical presentation can differ between children and adults [2]. Whereas the peak incidence of foreign body ingestion is between 6 months and 6 years [3], it is less frequent among adults and varies across populations [1]. Although nonbony food bolus is the most common in Western countries [4], in Asia, chicken and fish bones are more frequent [5]. Foreign body ingestion is slightly more common in males than females, with a ratio of 1.5 : 1 [6, 7]. Known risk factors of foreign body ingestion include extreme age [1], edentulism [8], maxillofacial trauma [9], psychoneurological deficit, and acute disorders of consciousness such as cerebrovascular accidents, alcohol intoxication, drug overdose, or general anesthesia [8].

Approximately 80% of all ingested foreign bodies pass spontaneously through the entire gastrointestinal tract [3, 4]. However, there is still significant morbidity and mortality associated with foreign body impaction [7]. Foreign body impaction usually occurs with physiologic constrictions, angulations, or stenosis of the gastrointestinal tract [11, 12]. The esophagus is the most common site of impaction, accounting for up to 70% of cases [5, 10]. Foreign body impaction in the small intestine is rare, and almost all reported cases occur in the terminal ileum [18, 19]. Finally, impaction in the large bowel is even less common, given its larger diameter compared to other areas of the gastrointestinal tract [9]. Thus, if any foreign body passes through the ileocecal valve, it generally passes through the colon without difficulty, unless there is a pathological stenosis or stricture such as with cancer.

The clinical presentation of foreign body impactions depends on the site of impaction and the presence of complications. Reported symptoms include dysphagia, odynophagia, chest pain, abdominal pain, or nausea/vomiting [20]. Additionally, surgical complications have been reported, including perforation, penetration of adjacent organs, bleeding, and obstruction [13–16].

Three management modalities have been described for foreign body ingestion and impaction: observation (wait and watch), endoscopy, and surgery [1]. Observation is possible when objects are small and do not provoke trauma to the gastrointestinal tract and the location is distal to the ligament of Treitz at the time of presentation [1]. In general, this strategy is more commonly used for uncomplicated lower gastrointestinal tract foreign bodies [1]. Endoscopy is the preferred modality for uncomplicated impaction, especially in the upper gastrointestinal tract [10]. Surgical intervention is warranted in complicated cases, regardless of the site of impaction [1]. Less than 1% of all cases require surgery [17].
